# Changing Perspectives on HDL: From Simple Quantity Measurements to Functional Quality Assessment

**DOI:** 10.1155/2021/5585521

**Published:** 2021-04-26

**Authors:** Himani Thakkar, Vinnyfred Vincent, Atanu Sen, Archna Singh, Ambuj Roy

**Affiliations:** ^1^Department of Biochemistry, All India Institute of Medical Sciences, New Delhi 110029, India; ^2^Department of Cardiac Biochemistry, All India Institute of Medical Sciences, New Delhi 110029, India; ^3^Department of Cardiology, All India Institute of Medical Sciences, New Delhi 110029, India

## Abstract

High-density lipoprotein (HDL) comprises a heterogeneous group of particles differing in size, density, and composition. HDL cholesterol (HDL-C) levels have long been suggested to indicate cardiovascular risk, inferred from multiple epidemiological studies. The failure of HDL-C targeted interventions and genetic studies has raised doubts on the atheroprotective role of HDL-C. The current consensus is that HDL-C is neither a biomarker nor a causative agent of cardiovascular disorders. With better understanding of the complex nature of HDL which comprises a large number of proteins and lipids with unique functions, recent focus has shifted from HDL quantity to HDL quality in terms of atheroprotective functions. The current research is focused on developing laboratory assays to assess HDL functions for cardiovascular risk prediction. Also, HDL mimetics designed based on the key determinants of HDL functions are being investigated to modify cardiovascular risk. Improving HDL functions by altering its composition is the key area of future research in HDL biology to reduce cardiovascular risk.

## 1. Introduction

The presence of water-soluble lipoproteins was reported for the first time by Michael Macheboeuf in 1929 when he isolated a class of proteins called alpha globulins, now recognized as high-density lipoprotein (HDL). In 1949, Gofman et al. proposed a new method for separation of lipoproteins from serum. They isolated HDL by ultracentrifugation and studied the association of lipoproteins with atherosclerosis [1]. Since then, HDL and its role in cardiovascular diseases (CVD) have been extensively studied.

HDL comprises heterogeneous particles varying in size, density, composition, and biological properties. Compared with other lipoproteins, HDL has the highest relative density (1.063-1.21 g/ml) and its size varies from 6.5 to 15 nm. In this review, we discuss the current evidence on the association of HDL with CVD.

## 2. The HDL Hypothesis

The protective role of high-density lipoprotein cholesterol in reducing the risk for CVD was reported for the first time in the 1950s [2]. Later in the 1980s, the landmark epidemiological study, Framingham Heart Study, reported lower incidence of coronary artery disease (CAD) in individuals with higher levels of HDL cholesterol [3]. The central proposed mechanism for the protective effect of HDL against atherosclerosis was the reverse transportation of cholesterol from the macrophages in the arterial wall back to the liver for removal from the body. Many epidemiologic studies conducted in different populations supported this inverse association of HDL levels and CAD, earning the title of “good cholesterol” for HDL cholesterol. Based on this epidemiological association in several studies [4, 5], clinical attempts to decrease CAD risk by way of pharmacologically increasing HDL-C levels were made. This would also help establish causality of the protective effect of HDL on CAD. The agents used were niacin and CETP inhibitors.

The Atherothrombosis Intervention in Metabolic Syndrome with Low HDL/High Triglycerides: Impact on Global Health Outcomes (AIM-HIGH) trial that studied the additional effect of niacin in patients with CVD and low HDL-C levels (<40 mg/dl in men and <50 mg/dl in women) in reducing cardiovascular disease involved 3414 high-risk patients who were receiving statin therapy. The trial was stopped early due to the lack of any additional clinical benefit of niacin over statin in reducing the incidence of CVD events. This was despite significant improvement in HDL cholesterol levels (HDL cholesterol level increased by 25.0% vs. 9.8% at 2 years in the niacin versus placebo group, *P* < 0.001, respectively) [6].

The Heart Protection Study 2-Treatment of HDL to Reduce the Incidence of Vascular Events (HPS2-THRIVE) trial was designed to assess additional benefit of niacin-laropiprant to statin-based therapy in 25,673 adults with known atherosclerotic vascular disease. The addition of niacin-laropiprant had no significant reduction in major vascular events as compared with placebo (13.2% and 13.7% of participants had a cardiovascular event, respectively; *P* = 0.29). Additionally, treatment with extended-release niacin-laropiprant increased the risk of serious adverse events [7, 8] including incident diabetes, gastrointestinal symptoms, musculoskeletal symptoms, skin disorders, infection, and bleeding.

Another class of drugs—cholesteryl ester transfer protein (CETP) inhibitors—increases HDL-C and decreases low-density lipoprotein cholesterol (LDL-C) and was tested to study the role of HDL cholesterol in reducing cardiovascular events. The first CETP inhibiting drug torcetrapib markedly increased HDL cholesterol levels in subjects with low HDL-C (106 percent relative to placebo (34 ± 5 mg/dl during the placebo phase to 70 ± 15 mg/dl)) and decreased LDL (17 percent relative to placebo (136 ± 24 mg/dl during the placebo phase to 114 ± 20 mg/dl)) cholesterol [9]. However, the clinical trial, ILLUMINATE, was stopped prematurely as torcetrapib had “off target” effects like rise in systolic blood pressure through stimulation of aldosterone synthesis [10]. In the ILLUMINATE study, majority of the torcetrapib treatment patients showed no regression of coronary atherosclerosis despite 72% increase in HDL-C [11]. Two other CETP inhibitors, dalcetrapib and evacetrapib, also failed to show significant reduction in CVD risk [12, 13]. The REVEAL (Randomized Evaluation of the Effects of Anacetrapib Through Lipid modification) study, addition of CETP inhibitor anacetrapib to intensive statin therapy, demonstrated a statistically significant reduction in composite end point of coronary death or myocardial infarction [14]. This was associated with 0.7 mmHg rise in BP in intervention arm. The first three trials with CETP inhibitors failed to show any reduction in risk of CVD events, as a result of which they were stopped. The manufacturers of anacetrapib did not file for regulatory approval to market the drug in view of probable anticipation of limited benefit/role of the drug in CVD management.

The cardioprotective hypothesis of HDL was also questioned by genome-wide association studies on genetic determinants of plasma lipid levels. Mendelian randomization studies identified single nucleotide polymorphism (SNP) in endothelial lipase gene (LIPG Asn396Ser) and 14 other SNPs that exclusively raise plasma HDL cholesterol levels. Polymorphism of LIPG gene and genetic score of 14 SNPs showed no association with risk of myocardial infarction as performed in prospective and case control studies [15].

Additionally, the traditional understanding of inverse relationship between HDL-C and CVD has also been challenged. According to the inverse linear relationship, those with extremely high HDL-C should be the most protected from CVD. However, recent prospective studies do not conform to this dictum. Extremely high HDL-C was associated with increased risk of mortality from CVD in both Japanese and Danish cohorts [16, 17]. The Danish cohort showed increased risk of all-cause mortality associated with extremely high HDL-C levels. Similar to these studies, a multicohort study also observed that CVD risk did not reduce further with HDL-C values higher than 90 mg/dl in men and 75 mg/dl in women [18].

Conflicting and inconsistent findings obtained from different clinical trials, genetic studies, and traditional epidemiological studies have led to the exploration of reasons for these differences. One of these is the measurement of HDL function rather than HDL-C level. The availability of technologies to explore different biological functions of HDL has spurred an interest in measuring HDL function and determining the most appropriate measure of HDL function instead of absolute HDL-C levels.

## 3. HDL Functions and CVD

**Figure 1:**
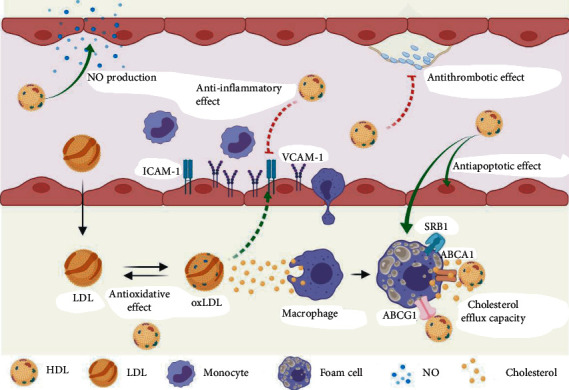
Antiatherogenic functions of HDL. NO: nitric oxide; HDL: high-density lipoprotein; LDL: low-density lipoprotein; oxLDL: oxidized LDL; SR-B1: scavenger receptor B1; ABCA1: ATP-binding cassette transporter A1; ABCG1: ATP-binding cassette transporter G1; ICAM-1: intercellular adhesion molecule 1; VCAM-1: vascular cell adhesion molecule 1.

It is increasingly evident that HDL particles have pleiotropic properties including cholesterol efflux capacity, antioxidant activity, anti-inflammatory activity, antithrombotic activity, and antiapoptotic activity. These contribute to the protective effect of HDL against atherosclerosis, and thus, singular HDL-C measurement may not be reflective of HDL role in health and disease.  Figure 1 summarizes different antiatherogenic functions of HDL.

### 3.1. Cholesterol Efflux Capacity (CEC)

HDL plays a crucial role in the initial step of reverse cholesterol transport, that is, the ability to accept cholesterol from peripheral cells including adipocytes, macrophages, and endothelial cells. This is considered to be the primary atheroprotective function of HDL. Membrane-bound lipid transporter ATP-binding cassette transporter A1 (ABCA1) transfers cellular cholesterol and phospholipid to lipid-free apolipoprotein A-I (apoA-I) forming discoidal pre-beta HDL. Besides ABCA1, two other proteins, ATP-binding cassette transporter (ABCG1) and scavenger receptor B1 (SR-B1), are also involved in the efflux of cholesterol to HDL particles. ABCA1 and SR-B1 efflux cholesterol to mature HDL from peripheral cells like macrophage and adipocytes [19]. Lecithin cholesterol acyltransferase (LCAT) activity by its transesterification property maintains the concentration gradient for cholesterol between peripheral cells and HDL; therefore, there is a unidirectional movement of cholesterol from cell to HDL thus facilitating reverse cholesterol transport [20]. Recent studies have demonstrated that the CEC of HDL also depends on the concentration of free cholesterol (FC) in HDL particles. As the movement of FC among lipid surfaces is reversible, the presence of high percentage of FC in HDL leads to the transfer of excess FC from HDL to cells *in vivo* [21].

The capacity of HDL to promote cholesterol efflux from macrophages was shown to have strong association with carotid intima media thickness independent of HDL-C levels [22]. Cholesterol efflux is mediated via different pathways to different HDL fractions; therefore, the efficiency of serum from an individual to take up cellular cholesterol is affected by the distribution and composition of HDL particles despite having similar levels of HDL cholesterol [23].

ABCA1-dependent serum CEC correlated inversely with pulse wave velocity, an index of arterial stiffness, independent of HDL-C serum levels in healthy individuals [24]. Cholesterol efflux capacity has also revealed an inverse correlation with noncalcified plaque burden, independent of traditional cardiovascular risk factors and HDL-C levels [25]. A large prospective study demonstrated that baseline CEC was significantly associated with incident cardiovascular events independent of HDL-C and apoA-I levels in the general population [26].

Studies performed in patients with metabolic syndrome or diabetes have also reported impaired HDL cholesterol efflux capacity to be an independent risk factor for the development of atherosclerosis [27–29]. Several studies have reported that impaired CEC is linked to the development of cardiovascular outcomes in patients in the context of inflammatory diseases like rheumatoid arthritis [30], systemic lupus erythematosus [31], and psoriatic arthritis [32].

Based on the observational studies that apoA-I is an efficient cholesterol acceptor that transports cholesterol from foam cells to the liver and aids regression of atherosclerosis, novel formulations of apoA-I were designed to induce cholesterol efflux from macrophages. One such recombinant molecule, CSL112, is a population of disc-shaped lipoprotein particles containing recombinant apoA-I and phosphatidylcholine [33]. In phase 2 clinical trial, 4 weekly infusions of CSL112 among patients with acute myocardial infarction reduced major adverse cardiovascular events without any significant alterations in liver or kidney functions [34]. CSL112 is currently being tested in a large-scale phase 3 cardiovascular clinical trial in patients with acute coronary syndrome (ACS) to test its efficacy [35].

However, a recent study utilizing Mendelian randomization found no association between apoA-I and incident CAD. Thus, the authors have suggested a noncausal role of HDL-CEC in the risk of CAD since apoA-I is the major determinant of CEC of HDL [36].

### 3.2. Antioxidative Activity

Oxidized LDL has been shown to be a major driving factor for the development of atherosclerosis [37]. The reactive, prooxidant molecules generated in response to cellular oxidative stress are responsible for the chemical modification of LDL. LDL is modified in two steps. The first step involves the formation of lipid hydroperoxides (LOOHs) which in turn propagate further oxidation generating free and core aldehydes and ketones that covalently modify *ε*-amino groups of lysine residues present in apoB which is called as oxLDL [38]. HDL protects LDL from oxidative stress induced by both one- and two-electron species by inhibiting accumulation of primary and secondary peroxidation products [39]. Hydroperoxides are transferred from LDL to HDL either spontaneously or by the process mediated by CETP. LOOHs are then removed from HDL via scavenger receptor class B1- (SR-B1-) mediated transfer to the liver [40].

Inhibition and reduction of LOOH depend on the chemical and physical properties of HDL. Transfer efficiency of hydroperoxides from LDL to HDL is governed by the fluidity of the HDL surface phospholipid monolayer [41]. Besides lipid content, protein composition of HDL affects its antioxidative activity. There are several proteins on HDL with antioxidative properties including apoA-I, apoA-II, apoA-IV, apoE, apoM, apoD, apoF, PON1, LCAT, and PAF-AH [42, 43].

Apolipoprotein A-I plays a crucial role in preventing the oxidation of LDL. Methionine residue at positions 112 and 148 in the apolipoprotein A-I reduces LOOH to their corresponding hydroxides and thereby terminates the chain reaction of lipid peroxidation. Tyrosine 115 is also involved in such redox reaction [44].

Apolipoprotein M secreted by the liver and kidney is found to be associated with HDL. It binds to oxidized phospholipids and enhances the antioxidative activity of HDL [45]. apoM also serves as a carrier of bioactive lipid, sphingosine-1-phosphate (S1P). The apoM-S1P complex contributes to the protective effects of HDL on endothelial cells by activating the antiatherosclerotic signalling pathways [46, 47]. LCAT has been shown to directly hydrolyze oxidized polar phospholipids [48]. HDL also carries lipophilic components like tocopherol, which make minor contribution to the HDL functionality in terms of its antioxidative function.

Paraoxonase 1 (PON1), an HDL-associated antioxidant enzyme, is capable of hydrolyzing lipid hydroperoxides and cholesterol ester hydroperoxides present on HDL and LDL [49]. PON1 has also been shown to have an ability to hydrolyze homocysteine-thiolactone to homocysteine. The natural substrates of PON1 are lactones, and due to the similarity of oxidized fatty acids with lactones, PON1 hydrolyzes fatty acids as well. Overexpression of PON1 in transgenic mice inhibits lipid hydroperoxide formation on HDL and thus protects HDL from oxidation and maintains its integrity [50].

Observational studies have reported that HDL antioxidant activity represents a strong and independent predictor of all-cause mortality in patients with acute coronary syndrome [51] and chronic heart failure [52] and critically ill patients [53] while one of the studies performed in cohort of renal transplant patients observed no association between HDL antioxidative activity and cardiovascular mortality [54].

### 3.3. Anti-Inflammatory Activity

During the genesis of atherosclerosis, inflammation induces endothelial cells to express adhesion molecules vascular cell adhesion protein 1 (VCAM-1), intercellular adhesion molecule 1 (ICAM-1), and E-selectins which leads to the adhesion of monocytes to endothelial cells. HDL inhibits the expression of the adhesion proteins and monocyte chemoattractant protein 1 (MCP-1) induced by tumor necrosis factor alpha (TNF-*α*) in endothelial cells [55] and macrophages. Studies done in vitro have shown that HDL and reconstituted HDL containing only apoA-I and phospholipids inhibit the expression of VCAM-1, ICAM-1, and E-selectin by human umbilical vein endothelial cells (HUVECs) in a concentration-dependent manner [56]. HDL prevents increase in intracellular reactive oxygen species and activation of proteasome and nuclear factor kappa B (NF-kappaB) triggered by ox-LDL in smooth muscle cells [57]. In monocytes, HDL reduces the expression of chemokines and their receptors via modulation of NF-kappaB and peroxisome proliferator-activated receptor gamma [58]. Apolipoprotein A-I prevents T cell-mediated activation of monocytes by inhibiting the production of TNF-alpha and interleukin 1. Apolipoprotein A-I also inhibits the function of activated neutrophils [59].

### 3.4. Antithrombotic Activity

HDL inhibits activation and aggregation of platelets, reduces von Willebrand factor levels, and enhances the activity of protein C and S [60]. In vitro, the inhibitory effect of HDL on platelet activation was demonstrated by incubation of isolated platelets with reconstituted HDLs and native HDLs [61]. Inhibition of platelet aggregation is dependent on SR-B1 and endothelial nitric oxide synthase (eNOS) [62].

### 3.5. Antiapoptotic Activity

In addition to possessing antioxidative and anti-inflammatory activities, HDL inhibits apoptosis of macrophages and endothelial cells induced by oxidized LDL [63]. HDL protects macrophages from apoptosis induced by oxidized LDL by promoting the efflux of cholesterol [64]. Small dense HDL3 subfraction protects endothelial cells from apoptosis and oxidative stress induced by oxLDL by reducing the release of cytochrome c, inhibiting caspase-3 activity, and preventing degradation of DNA [65]. Interaction of HDL with ABCA1 and ABCG1 in macrophages activates the antiapoptotic signalling pathway via AKT and NF-kappaB [66]. HDL also exerts an antiapoptotic effect on pancreatic beta cells leading to decrease in progression of diabetes mellitus [67].

Though currently it is not clear which particular functionality of HDL is cardioprotective, meta-analyses have reported a negative association of cholesterol efflux, antioxidant, and anti-inflammatory capacities with major adverse cardiovascular events (MACE) and all-cause mortality [68–70].

## 4. Assessment of HDL Functions

Given the multiplicity and complex attributes of the pleiotropic functions of HDL, many different assays have been developed to assess their utility as a marker for cardiovascular health and delineate the quantum of their respective contributions to cardiovascular health and disease. However, a composite measure of HDL function still eludes researchers and clinicians. The current measures of the various facets of HDL function discussed are as follows.

### 4.1. Cholesterol Efflux Capacity

The cholesterol efflux assay is aimed at quantifying the efflux of cholesterol from cultured cells to an acceptor particle or to plasma. Cholesterol efflux capacity of HDL is measured *in vitro* using a donor and a cholesterol acceptor. The protocol for efflux assay used in different laboratories differs by type of cell, acceptor, efflux time, and specificity of the transporters [22, 71, 72]. In order to measure HDL's ability to take up cholesterol, whole serum, apoB-depleted serum, or isolated HDLs are used as acceptors. apoB-depleted serum is preferred over other HDL sources as removing LDL also reduces the exchange of cholesterol and it prevents shedding of apolipoproteins. Different macrophage cell lines like J774, THP-1, and RAW 264 have been used as donors. Cholesterol is released from macrophages either by aqueous diffusion or through transporters (ABCA1, ABCG1, and SR-B1). Cell lines are treated with some inducers in order to increase the expression of transporters or to identify the contribution of a specific transporter to the efflux. Since cholesterol efflux from cells depends on the intracellular lipid metabolism, acyl-coenzyme A:cholesterol acyltransferase (ACAT) inhibitors are used in some studies to prevent formation of cholesterol esters [73].

Initial cholesterol efflux assays employed radiolabelled (^3^[H]) cholesterol probes to measure HDL cholesterol efflux capacity. To avoid the use of radioisotopes in the assay, fluorescently labelled cholesterol that is boron dipyrromethene difluoride cholesterol probe (BODIPY-cholesterol) has been used as an alternative. BODIPY-based CEC assays have shown significant agreement with [(^3^)H] cholesterol-based CEC assay [74].

Although the cholesterol efflux assay has great potential to be used for cardiovascular risk assessment, there are several limitations to the assay in its current form. As the assay is cell based, it is time consuming and labor intensive. The current CEC assay is difficult to apply in routine clinical practice because of the lack of standardized protocol. Currently, cell-free assay systems (liposome and antibody based) are being explored to assess CEC of HDL in clinical settings [75, 76].

### 4.2. Antioxidative Activity

HDL antioxidant function assessment involves cell-free assays of HDL oxidation [77–79]. The cell-free assays utilized fluorescent molecules like dichlorodihydrofluorescein diacetate (DCF-DA) and dihydrorhodamine (DHR) molecules to study the ability of HDL to prevent the formation of oxidative products. Suppression of oxidation of the fluorescent molecules reflects the antioxidative activity of HDL. The short self-life of the fluorescent probes limits the clinical utility of this assay to evaluate HDL antioxidative activity and needs improvisations.

The activity of HDL-associated antioxidative enzymes (PON1, glutathione peroxidase) is also measured. Arylesterase and paraoxonase activity of PON1 are measured using phenyl acetate and paraoxon as substrate by spectrometry [80]. The ability of HDL to inhibit LDL oxidation [81], expression of monocyte chemoattractant protein 1 (MCP1), and adhesion molecules are used to determine HDL anti-inflammatory index. The application of HDL anti-inflammatory index as a maker for HDL function in routine practice is limited due to low reproducibility of the approach.

### 4.3. Endothelial eNOS and VCAM-1/ICAM-1 Assay

Endothelial protective effects of HDL are analyzed by measuring the production of nitric oxide by electron spin resonance spectroscopy or by fluorescence-based techniques in cell system. NO production is also analyzed by peripheral arterial tonometry (Endo-PAT) [82] which measures nitric oxide-dependent vasodilation in large vessels. Test based on electron spin resonance spectroscopy is also used in some clinical laboratories to determine NO production ability of HDL [83]. Vasoprotective activity of HDL is evaluated by measuring the expression of adhesion molecules (VCAM-1and ICAM-1) by western blot, real-time PCR [84], ELISA [85], or flow cytometry [86]. The assay needs to be validated in large-scale studies for its use in clinical settings.

### 4.4. Antiapoptotic Activity

For antiapoptotic property of HDL on endothelial cells and pancreatic beta cells, expression of caspase-3 (as marker of apoptosis) and molecules involved in the signalling pathway is analyzed by western blot or by real-time PCR. The requirement of cultured cells limits the assessment of antiapoptotic activity of HDL to research usage only currently.

## 5. HDL Structure-Function Relationship

The HDL particle is a complex of proteins, lipids, microRNAs (miRNA), and metabolites. It has high protein to lipid ratio in which apolipoprotein A-I accounts for 70% of the total protein of the particle. Apolipoprotein A-II is the second most abundant protein. Besides these two proteins, HDL particle comprises more than 90 proteins and 200 lipids. The lipid component of HDL particle comprises cholesteryl esters (CE), free cholesterol (FC), triglycerides (TG), and phospholipids (PL). PL and FC constitute the surface lipid monolayer, while CE and TG form the hydrophobic lipid core. HDL particles differ in composition and size and exhibit a range of atheroprotective properties, and these properties are exerted by the different protein and lipid components of HDL [87, 88]. With recent research and evidence available about HDL functionality, it is now being asserted that the quality rather than quantity of HDL is more relevant for its atheroprotective activity and the structural basis for the functional aspects of HDL is being evaluated [89].

### 5.1. HDL Proteome

HDL carries a large number of proteins which provide the structural and functional characteristics unique to HDL particles. HDL proteins are divided into subgroups based on functionality and include apolipoproteins, enzymes, lipid transfer proteins, proteinase inhibitors, acute phase response proteins, and complement components [90–93]. Around 110 proteins associated with HDL have been identified using different approaches. Majority of the studies have used ultracentrifugation to separate HDL from the serum prior to proteome analysis.

Since HDL functionality is influenced by its associated proteins, recent research is focused on identifying HDL-associated proteins as surrogate markers for HDL functions. Moreover, HDL proteins associated with its functions can serve as viable targets for developing drugs that could lower CVD risk.

### 5.2. HDL Lipidome

The lipid component of HDL particle comprises cholesteryl esters (CE), free cholesterol (FC), triglycerides (TG), and phospholipids (PL). PL and FC constitute the surface lipid monolayer, while CE and TG form the hydrophobic lipid core. Phospholipids quantitatively predominate in the HDL lipidome accounting for 36%–40% of total lipid. More than 200 lipid molecules have been identified on HDL isolated from healthy normolipidemic individuals [94, 95].

HDL lipidome is significantly altered in pathological conditions like dyslipidemia, coronary artery disease, and hypertension. Nuclear magnetic resonance (NMR) analysis has shown alteration in the composition of HDL fraction in subjects with coronary artery disease, with higher percentage of triglyceride and lower percentage of cholesterol esters, phosphatidylcholine, and sphingomyelin [96]. HDL phospholipid composition affects SR-B1-mediated cholesterol efflux, which thereby impacts the process of reverse cholesterol transport [97]. HDL is a major carrier of sphingosine-1-phosphate which plays a key role in endothelial functions and the cardiovascular system [98]. HDL lipidome studies using mass spectrometry or NMR techniques are cumbersome to set up, limiting the translational value of these studies. Unlike HDL proteome, it is difficult to target HDL lipidome for modulation of cardiovascular risk.

Distinct structural or biochemical changes in HDL particles that lead to alteration of HDL functions have been reported in various studies. Some changes have shown a clear relationship with alterations in HDL functionality, while the mechanism underlying some others is yet to be completely elucidated. Structural changes include the alteration in composition of the HDL-associated proteins and lipids. Proteomic changes like an increase in the content of serum amyloid A1, serum amyloid A2, and alpha-1 antitrypsin on HDL and a decrease in the levels of apoA-I and paraoxonase 1 lead to the formation of dysfunctional HDL and attenuation of its atheroprotective functions [99–101]. A higher sphingomyelin to phosphatidylcholine ratio contributes to a diminished HDL antioxidative activity by altering the rigidity of the surface monolayer of HDL [102].

Biochemical changes like posttranslational modifications of the structural components also impact the protective properties of HDL. Myeloperoxidase (MPO), a major constituent of artery wall macrophages, induces MPO-catalyzed nitration, chlorination, and oxidation of apoA-I [103, 104]. MPO-mediated oxidative modification of methionine residue of apoA-I at position 148 was observed in subjects with coronary artery disease and was associated with decreased cholesterol efflux capacity [105]. Modification at tyrosine residue of apoA-I also showed impaired ABCA1-dependent cholesterol transport [106]. The glycation of apoA-I alters the conformation of apoA-I in regions that are critical for LCAT activation, reducing the cholesterol efflux capacity and the anti-inflammatory activities of HDL [107, 108].

### 5.3. HDL Subclasses

**Figure 2:**
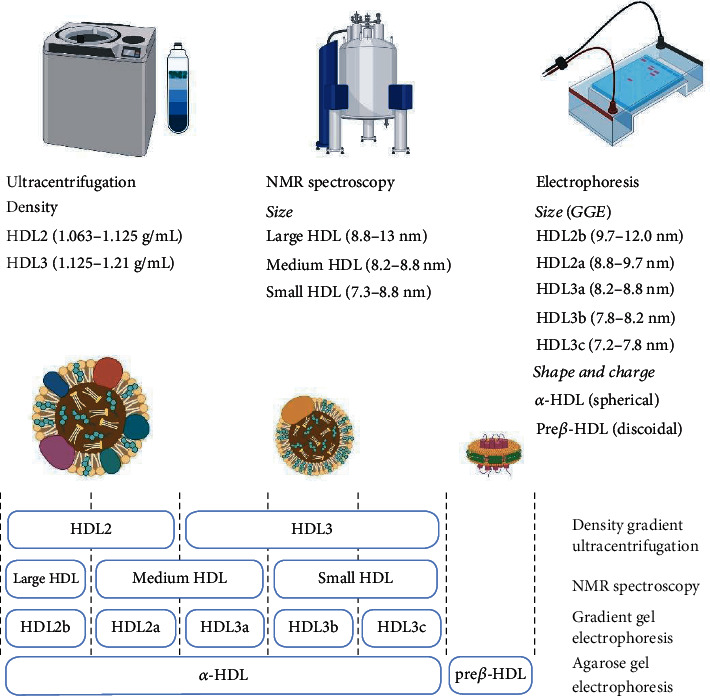
HDL subclasses characterized by different methods of separation. NMR: nuclear magnetic resonance; GGE: gradient gel electrophoresis.

Human HDL particles are highly heterogeneous consisting of several subclasses differing in density, size, lipid composition, and protein composition.  Figure 2 shows different subclasses of HDL characterized by different methods of separation.

It has been suggested that HDL subclasses, including the different measures of HDL particle heterogeneity, are better markers for CVD in comparison to static measures of HDL mass like cholesterol content. In comparison to HDL cholesterol, the profile of HDL particles showed stronger association with atherosclerosis [109]. In a cross-sectional analysis performed on the multiethnic study of atherosclerosis (MESA) cohort, small- and medium-sized HDL particles assessed using NMR were found to be strongly and inversely associated with carotid intima thickening [110]. A prospective study has observed that baseline HDL3-C levels were an independent protective factor against arterial stiffness, while no association was observed between HDL2-C and carotid pulse wave velocity [111]. HDL particle size has also been shown to determine the functions of HDL like cholesterol efflux capacity [112].

The distribution of proteins varies across the HDL fractions. Small and dense HDL3 has higher protein content which was confirmed by mass spectrometric analysis of HDL subfractions. There are some proteins which are specifically present on HDL3 particles like apoJ, apoL-1, apoF, PON1/3, PLTP, and PAF-AH [113]. HDL3 fraction has greater number of HDL-associated enzymes: LCAT, PON1, and PAF-AH. apoE, apoC-I, and apoC-III are present on larger HDL2. Specific protein-protein interactions, facilitated by lipids, account for heterogeneity in the complement of HDL proteins which provides distinct functionalities to HDL fractions. These subfractions play a role not only in lipid metabolism but also in acute phase response, innate immune response, and plaque stability [91].

### 5.4. HDL Particle Number and Mean Particle Size

Concentration (number) of HDL particles in circulation and mean size of HDL particles are emerging predictors of CVD risk. HDL particle numbers are quantified through nuclear magnetic resonance (NMR) spectroscopy, ion mobility assay, or gradient gel electrophoresis [114]. In MESA cohort and JUPITER trial (Justification for the Use of statins in Prevention: an Intervention Trial Evaluating Rosuvastatin), elevated number of HDL particles was associated with reduced risk of incident CVD [115]. Mean HDL size measured using NMR or ion mobility measurements is an integrative measure of HDL heterogeneity. Mean HDL size shows an inverse association with CVD risk [115, 116]. HDL particle size has also been demonstrated to impact HDL functions like CEC and paraoxonase activity [117].

## 6. Conclusion

The future of HDL as a biomarker for CVD is rapidly evolving. Simultaneously, new information regarding the structural and functional complexity of HDL is emerging. Thus, there is an emerging consensus that HDL structural components and functional aspects may be better predictors of CVD risk than static mass of HDL measured through HDL-C. Recent research findings advocate the use of HDL functions like CEC levels as the predominant therapeutic targets rather than HDL cholesterol mass. This could be the norm in the future clinical practice with the advent of standardized assays for HDL functions like CEC. Additionally, research to identify HDL components that manifest HDL functionality and whose assessment is widely amenable in clinical setups may be in wider use. Thus, an assay for composite measure of HDL function that is adaptable for clinical setup should be the goal for future HDL research and should study its impact on the risk of CVD. Also, HDL as a therapeutic agent for primary and secondary prevention of CVD is emerging and being tested in clinical trials and charters a path different from the earlier failures of HDL-C-elevating drugs. Studies are focusing on improving the HDL functions in individuals with supplementation of recombinant HDL or HDL components like recombinant apoA-I. Understanding the complex nature of HDL and its role as a protective agent, biomarker, and therapeutic target in CVD remains an exciting area of research.
